# Supplementary material for: Attention to Speech and Music in Young Children with Bilateral Cochlear Implants: A Pupillometry Study
Source: J Clin Med. 2022 Mar 21;11(6):1745. doi: 10.3390/jcm11061745 (PMC8956090; doi:10.3390/jcm11061745)

S1. Initial model description

Using the function buildmer in the package buildmer, we have initially run the tested the fit of the model with the interaction of the main factors and the sound pressure level as an additional random intercept (mean ~ 1 + group + noise + signal + group*noise*signal + (1 | subj) + (1|sound)) using g)lm, gam, or (g)lmer objects. The maximal feasible model was the following: mean ~ 1 + group + noise + signal + (1|subj); all higher models failed to converge. This model was then tested with the permutation linear mixed-effects model, so that we could easily obtain robust p-values. The advantage of these permutation p-values is that we could run multiple such models without requiring any Bonferroni correction.

Model Summary:
Linear mixed model fit by REML ['lmerMod']
Formula: mean ~ 1 + group + noise + signal + (1|subj)

REML criterion at convergence: -69.2
Scaled residuals:

Min	1Q	Median	3Q	Max
-3.7447	-0.6182	-0.0111	0.6192	3.2935

Random effects:

Groups	Name	Variance	Std.Dev.
subj	(Intercept)	0.01525	0.1235
Residual	0.04298	0.2073	

Number of obs: 478, groups: subj, 28

Fixed effects:

	Estimate	Std.Error	t-value	Pr(>|t|)	
(Intercept)	0.18829	0.04123	4.567	<2e-16	***
groupNH.group	0.11697	0.05162	2.266	<2e-16	***
noiselow.noise	-0.01831	0.02318	-0.790	0.203	
noisehigh.noise	0.05057	0.02341	2.160	0.003	**
signalspeech	0.04326	0.02369	1.826	0.013	*
signalmusic	0.06362	0.02327	2.734	<2e-16	***

Signif. codes: 0 '***' 0.001 '**' 0.01 '*' 0.05 '.' 0.1 ' ' 1

Correlation of Fixed Effects:

	(Intr)	grpNH	nslw.n	nshgh	sgnlsp
groupNH.grp	-0.627				
noiselow.ns	-0.274	0.008			
noisehgh.ns	-0.279	-0.006	0.490		
signalspech	-0.285	0.000	-0.026	0.012	

signalmusic	-0.290	-0.015	-0.008	0.024	0.512

Type III Analysis of Variance of the model with Satterthwaite's method

	npar	SumSq		MeanSq	F-value	Pr(>F)
group	1	0.2348771	0.2348771	5.464434	0.01940734	
noise	2	0.3684189	0.1842095	4.285647	0.01376471	
signal	2	0.3319152	0.1659576	3.861016	0.02104660	

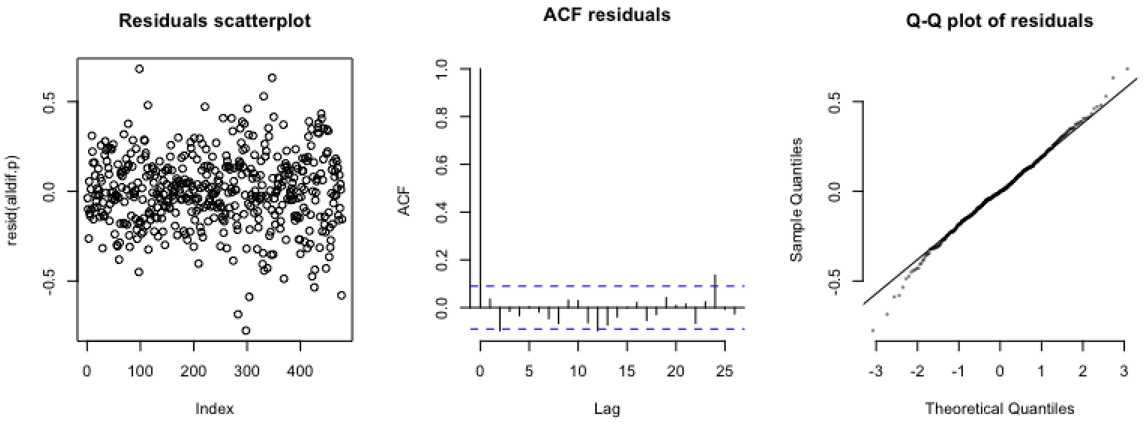

(**A**) (**B**) (**C**)

Figure S1. (**A**) Correlation plot of the residuals of the model initial model. (**B**) Autocorrelation plot of the residuals of m1. (**C**) Q-Q plot of the residuals of the initial model.

S2. Permutation-based time-course analysis

The following permutation analyses were conducted separately for each group:

Analysis of the differences between various noise conditions during speech stimuli:

```
Speech.in.noise.CI.group   ←   clusterperm.lmer(pupil ~ noise + (1|event),
        data=subset(group == "CI.group" & signal == "speech"), series.var=~time)
Speech.in.noise.NH.group ←   clusterperm.lmer(pupil ~ noise + (1|event),
        data=subset(group == "NH.group" & signal == "speech"), series.var=~time)
```

Analysis of the differences between various noise conditions during music stimuli:

```
Music.in.noise.CI.group   ←   clusterperm.lmer(pupil ~ noise + (1|event),
        data=subset(group == "CI.group" & signal == "music"), series.var=~time)
Music.in.noise.NH.group ←   clusterperm.lmer(pupil ~ noise + (1|event),
        data=subset(group == "NH.group" & signal == "music"), series.var=~time)
```

Following Maris & Oostenveld (2007), the p-values corresponding to the cluster-mass test were computed for each time-sample. Although this could already comprise the entirety of the analysis, we used an approach more common fields that use time-course data, which is to use the time samples selected with the cluster-mass test as an input for our actual analysis. Thus, for each analysis, the time-window from the first till the last significant time-sample was selected as an input for our actual analysis, a linear mixed-effects model. We averaged our data along the selected window and then run a permutation linear mixed-effects model to obtain robust p-values.

Speech.in.noise.CI.group:

Linear mixed model fit by REML ['lmerMod']
Formula: spin.ci.mean ~ 1 + noise + (1|subj)

REML criterion at convergence: 0.4
Scaled residuals:

	Min	1Q	Median	3Q	Max
-2.72790	-0.54685	0.00106	0.66116	2.13918	

Random effects:

Groups	Name	Variance	Std.Dev.
subj	(Intercept)	0.01085	0.1042
Residual	0.04461	0.2112	

Number of obs: 74, groups: subj, 14

Fixed effects:

	Estimate	Std.Error	t-value	Pr(>|t|)	
(Intercept)	0.38443	0.05189	7.409	<2e-16	***
noiselow.noise	-0.07686	0.05958	-1.290	0.070	.

noisehigh.noise	-0.08237	0.06242	-1.320	0.062	.

Signif. codes: 0 ‘***’ 0.001 ‘**’ 0.01 ‘*’ 0.05 ‘.’ 0.1 ‘ ’ 1

Correlation of Fixed Effects:

	(Intr)	nslw.n
noiselow.ns	-0.599	
noisehgh.ns	-0.562	0.487

Type III Analysis of Variance of the model with Satterthwaite's method

	npar	SumSq	MeanSq	F-value	Pr(>F)
noise	2	0.1022162	0.05110812	1.145638	0.3180211

Music.in.noise.CI.group:

Linear mixed model fit by REML ['lmerMod']
Formula: muin.ci.mean ~ 1 + noise + (1 | subj)
Data: data

REML criterion at convergence: 28.6

Scaled residuals:

Min	1Q	Median	3Q	Max
-1.80396	-0.62069	-0.07954	0.65678	2.57829

Random effects:

Groups	Name	Variance	Std.Dev.
subj	(Intercept)	0.01973	0.1405
Residual	0.06417	0.2533	

Number of obs: 78, groups: subj, 13

Fixed effects:

	Estimate	Std.Error	t-value	Pr(>|t|)	
(Intercept)	0.21356	0.06392	3.341	<2e-16	***
noiselow.noise	0.06361	0.06886	0.924	0.138	
noisehigh.noise	0.03930	0.07332	0.536	0.366	

Signif. codes: 0 ‘***’ 0.001 ‘**’ 0.01 ‘*’ 0.05 ‘.’ 0.1 ‘ ’ 1

Correlation of Fixed Effects:

	(Intr)	nslw.n
noiselow.ns	-0.566	
noisehgh.ns	-0.523	0.490

Type III Analysis of Variance of the model with Satterthwaite's method

	npar	SumSq	MeanSq	F-value	Pr(>F)
noise	2	0.05534035	0.02767017	0.4312122	0.649721

Music.in.noise.NH.group:

Linear mixed model fit by REML ['lmerMod']

Formula: muin.nh.mean ~ 1 + noise + (1 | subj)

REML criterion at convergence: 10.9

Scaled residuals:

	Min	1Q	Median	3Q	Max
-2.50937	-0.51500	-0.07614	0.61248	2.56408	

Random effects:

Groups	Name	Variance	Std.Dev.
subj	(Intercept)	0.01278	0.1130
Residual	0.05158	0.2271	

Number of obs: 91, groups: subj, 14

Fixed effects:

	Estimate	Std.Error	t-value	Pr(>|t|)	
(Intercept)	0.30312	0.05036	6.020	<2e-16	***
noiselow.noise	0.05736	0.05843	0.982	0.154	
noisehigh.noise	0.24514	0.05810	4.219	<2e-16	***

Signif. codes: 0 '***' 0.001 '**' 0.01 '*' 0.05 '.' 0.1 ' ' 1

Correlation of Fixed Effects:

	(Intr)	nslw.n
noiselow.ns	-0.528	
noisehgh.ns	-0.529	0.463

Type III Analysis of Variance of the model with Satterthwaite's method

	npar	SumSq	MeanSq	F-value	Pr(>F)
noise	2	0.9803567	0.4901784	9.504038	7.455015e-05

Figure S2A. Time windows defined by the cluster mass statistic for the permutation-based analysis of speech in noise in the CI group.

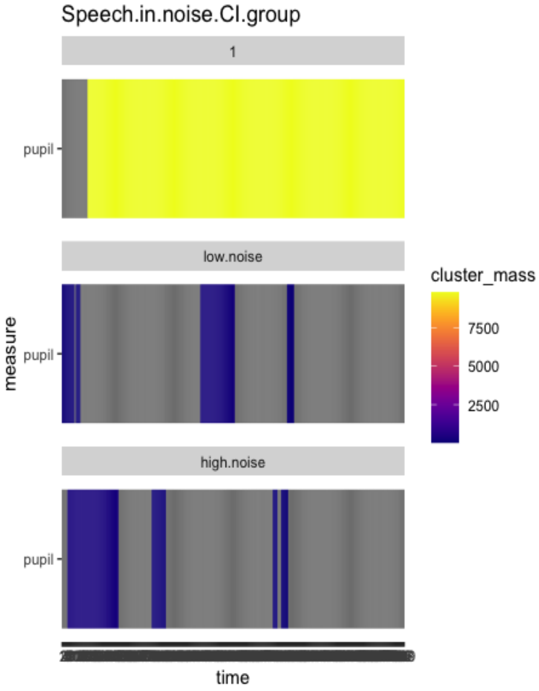

Figure S2B. Time windows defined by the cluster mass statistic for the permutation-based analysis of speech in noise in the NH group.

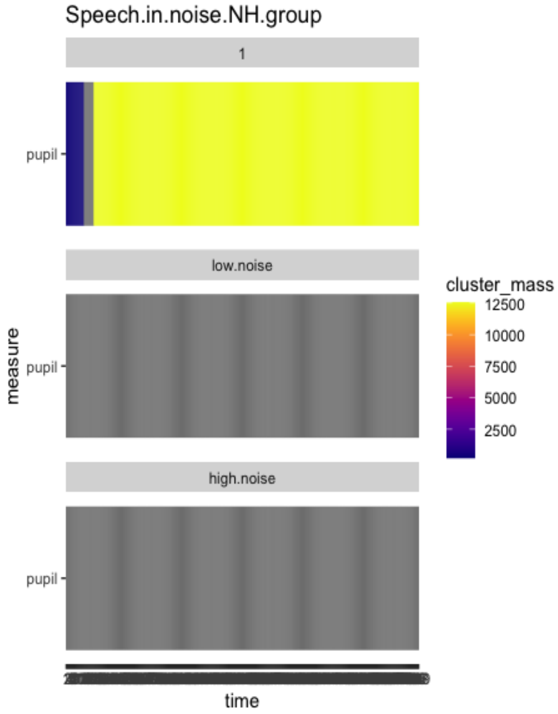

Figure S2C. Time windows defined by the cluster mass statistic for the permutation-based analysis of music in noise in the CI group.

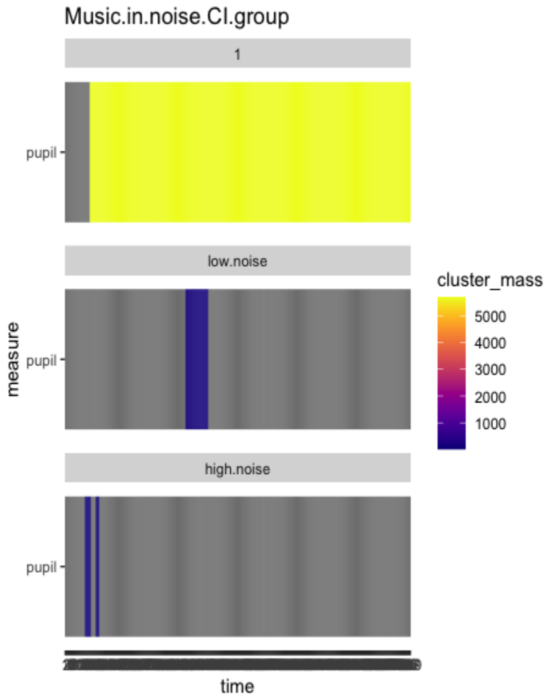

Figure S2D. Time windows defined by the cluster mass statistic for the permutation-based analysis of music in noise in the NH group.

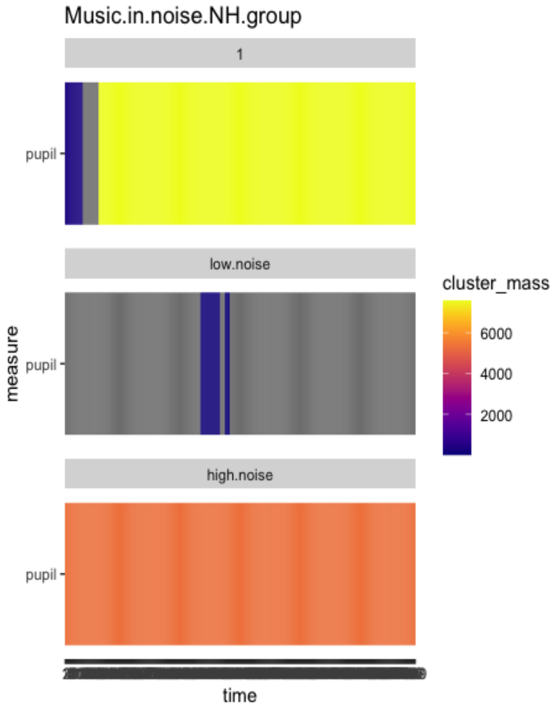

Supplement: Supplementary file 1 [file jcm-11-01745-s001.zip › jcm-1585535-supplementary.pdf]
